# Large size self-assembled quantum rings: quantum size effect and modulation on the surface diffusion

**DOI:** 10.1186/1556-276X-7-520

**Published:** 2012-09-24

**Authors:** Cunzhu Tong, Soon Fatt Yoon, Lijun Wang

**Affiliations:** 1 State Key Laboratory of Luminescence and Applications, Changchun Institute of Optics, Fine Mechanics and Physics, Chinese Academy of Sciences, Changchun, 130033, China; 2 School of Electrical and Electronic Engineering, Nanyang Technological University, Singapore, 639798, Singapore

**Keywords:** Quantum rings, Self-assemble, Quantum size effect, Surface diffusion

## Abstract

We demonstrate experimentally the submicron size self-assembled (SA) GaAs quantum rings (QRs) by quantum size effect (QSE). An ultrathin In_0.1_ Ga_0.9_As layer with different thickness is deposited on the GaAs to modulate the surface nucleus diffusion barrier, and then the SA QRs are grown. It is found that the density of QRs is affected significantly by the thickness of inserted In_0.1_ Ga_0.9_As, and the diffusion barrier modulation reflects mainly on the first five monolayer . The physical mechanism behind is discussed. The further analysis shows that about 160 meV decrease in diffusion barrier can be achieved, which allows the SA QRs with density of as low as one QR per 6 μm^2^. Finally, the QRs with diameters of 438 nm and outer diameters of 736 nm are fabricated using QSE.

## Background

Quantum rings (QRs), being viewed as the artificial benzene analogs, have attracted the increasing interests due to their novel magnetic and optoelectronic properties [1-4] and the potential applications as high performance THz detectors [5,6], solar cells [7], lasers [8], and single photon sources [9]. As a kind of low dimensional nanostructures, the physical properties of QRs are governed by their configuration. Hence, manipulate atoms to control the size and shape of QRs are strongly pursued. The conventional approaches including the different growth temperature [10], the annealing time [11], the arsenic pressure used in the annealing [12], and partially capping growth [13] had been applied to fabricate the different shape self-assembled (SA) QRs. However, there are few investigations from the surface energy or diffusion barriers point of view which also affects the atom diffusion from the well-known Arrhenius relation of diffusion coefficient.

Surface energy and diffusion barriers had been demonstrated to be sensitive to the thickness of ultrathin epitaxy layer on the surface [14-17] due to the quantum size effect (QSE). In the ultrathin epitaxy layer, the energy levels are discrete and the surface free energy of film is dependent on the position of these discrete levels. It has been found that the diffusion barrier is sensitive to the number of atom layers in the film [15], and the density and energy stability of metallic nanostructures also show a bi-layer oscillation behavior [14]. Hence, QSE has been recognized as a strong driving force for self-assembly and used to select certain preferred sizes and geometry of the nanostructures in the growth process [15]. In this paper, we demonstrated the submicron size GaAs QRs by modulating the surface energy, which were achieved by the QSE of InGaAs nanolayer. It was found that the size and density of QRs can be controlled by changing the deposition thickness of low surface energy layer. The dependence of diffusion barrier on the thickness of InGaAs is studied and the mechanism behind is discussed.

## Methods

The QRs were grown using a Riber 32P solid-source molecular beam epitaxy (MBE) system. After oxide desorption, a 300-nm GaAs buffer layer was deposited on the semi-insulating GaAs (100) wafers at 580°C. Then, the substrate was cooled down to 480°C, the In_0.1_ Ga_0.9_As layer was grown with the thickness regime from 1 to 14 monolayer (ML). After that, the arsenic valve was closed for 3 min. Subsequently, gallium atoms equivalent to form 10 ML GaAs were supplied to the substrate surface to form the gallium droplets at the setting temperature. The growth rate was 1 ML/s. Then, the gallium droplets were annealed in the As4 ambient with the arsenic pressure of 1 × 10^−6^ Torr, which is equivalent to the V/III ratio of 16. Annealing time is 100 s to make sure all the gallium atoms crystallize to form the GaAs [11]. The growth temperature of QRs is 480°C for the different thicknesses of In_0.1_ Ga_0.9_As. The QRs with different growth temperatures on the 5-ML In_0.1_ Ga_0.9_As are also grown to analyze the surface diffusion. After quenching, the sample was taken out from the MBE chamber for analysis using a Shimadzu SPM 9500 atomic force microscopy (AFM) (Shimadzu Scientific Instruments, Japan) in tapping mode.

## Results and discussions

**Figure 1:**
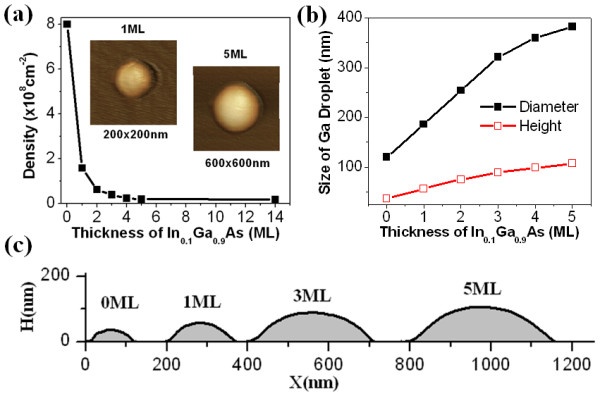
**Gallium droplet characteristics.** (**a**) Droplet density as a function of In_0.1_ Ga_0.9_As thickness; inset shows the AFM image of single gallium droplet. (**b**) The diameter (solid squares) and height (hollow squares) of gallium droplets. (**c**) The cross profile of gallium droplets.

Figure 1 shows the evolution of droplet density, size, and shape on the underneath In_0.1_ Ga_0.9_As thickness grown at 480°C. As shown in Figure 1a, the droplet density decreases significantly with the increase in the thickness of In_0.1_ Ga_0.9_As layer, which reflects that the inserted InGaAs layer changes the surface diffusion barrier of gallium atoms significantly. When the thickness of In_0.1_ Ga_0.9_As is above 3 ML, the surface density of droplet decreases over ten times, and then the variation in diameter and height becomes gentle (Figure 1b). The density is as low as 1.7 × 10^7^ cm^−2^ or about one droplet per 6 μm^2^ for a InGaAs layer of 14 ML. Compared with the surface density of droplet grown directly on the GaAs (approximately 8 × 10^8^ cm^−2^), the density reduces almost 47 times. Note that the density of QRs is same as that of the droplets due to the formation of QRs achieved by annealing the droplets. This extremely low density can be beneficial for the fabrication of single QR device as well as single quantum dot (QD) device because the similar droplet epitaxy approach is also used to fabricate the QDs [4]. Figure 1c shows the measured cross-section of gallium droplets; the top view AFM images are shown in the inset of Figure 1a, which reveals that the droplet volume increases with the decrease in the density, and the shape is almost always circle. The difference of droplet size in the two crystal directions of GaAs (001), i.e. [1-10] and [110] direction, is less than 1.1%, reflects that the gallium atom diffusion on the InGaAs in the vacuum is almost independent on the crystal direction. Note that the formation of gallium droplets discussed in above analysis is done in the vacuum without arsenic ambient, so the above results are not in conflict with the conclusion that Ga diffusion on the GaAs (001) surface is strongly anisotropic [18] in the stable arsenic ambient. 

**Figure 2:**
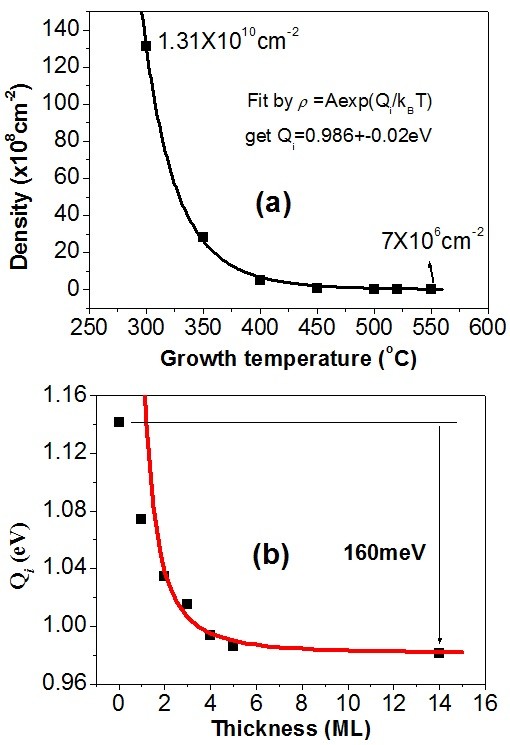
**Droplet density and best fit results.** (**a**) The surface density of QRs grown on 5-ML In_0.1_ Ga_0.9_As at different growth temperatures. (**b**) The diffusion barrier versus the thickness of In_0.1_ Ga_0.9_As.

To obtain quantitavely the surface diffusion barrier modulated by the inserted InGaAs layer, the following analysis is presented. The size and shape of QRs grown by droplet epitaxy are determined mainly by the characteristics of droplets because it is the diffusion of atoms in the droplets that forms the QRs. If the surface coverage is fixed, the size of the droplets will be inversely proportional to their density, which can be successfully described by mean-field rate equation theories [19-22]. From these theories, the surface density of stable islands *ρ* is determined by the surface diffusion barrier and can be scaled as $\rho \propto {\left(D/F\right)}^{-i/\left(i+2\right)}$ for a fixed coverage, where *D* is the adatom diffusion coefficient, *F* the incoming flux, and *i* the size of the critical nucleus. For the experimental results shown in Figure 1, the incoming flux is fixed, so the gallium droplets density can be expressed as:

(1) $$\rho =A\exp\left(Q_{i}/k_{\text{B}}T\right)\text{,}$$

where *A* is a constant, *k*_B_ and *T* are the Boltzmann constant and temperature, respectively. *Q*_*i*_ is the nucleus diffusion barrier and is equal to $\frac{i}{i+2}Q$, where *Q* is the diffusion energy barrier. We grew the droplets on the 5-ML In_0.1_ Ga_0.9_As at different temperatures The droplet density as a function of growth temperature is plotted in Figure 2a. The density reduces from 1.31 × 10^10^ cm^−2^ at 300°C to 7 × 10^6^ cm^−2^ at 550°C, which is about 187 times decreased. So, the significant variation of density is due to the increase of temperature from 300°C to 550°C that is equivalent to 54.5% decrease in the nucleus diffusion barrier. From the best fit by Equation 1, it is obtained that the *Q*_*i*_ is approximately 0.986 eV. From that, we can obtain the values of nucleus diffusion barrier *Q*_*i*_ for different thicknesses of InGaAs according to Equation 1 due to the same growth temperature, which are plotted in Figure 2b. As can be seen, the diffusion barrier of gallium decreases at first rapidly and then gradually with the increase in the thickness of In GaAs. The maximum decrease of diffusion barrier is about 160 meV, and the nucleus diffusion barrier on the GaAs and the thick In_0.1_ Ga_0.9_As is 1.141 and 0.981 meV, respectively, which reveals that the maximum decrease of nucleus diffusion barrier achieved by QSE is about 14%. Above 5 ML, the influence of epitaxial thickness on the diffusion barrier becomes almost negligible; in other words, the modulation on the surface diffusion energy barrier by the QSE of the deposited InGaAs layer reflects mainly on the first 5 ML. The underlying physical mechanisms giving rise to the QSE on surface diffusion barrier might be complex. The experiment [23] has revealed that the highest occupied quantum-well (QW) states near Fermi level will likely affect the adatom surface binding energies and diffusion barriers. A scanning tunneling spectroscopy study [24] also shows that the position of the conduction-band minimum and the valence-band maximum relative to the Fermi level affects the surface potential barrier of InAs/GaAs material. The energy levels in QWs are inversely proportional to the square of QW width [25,26], and the Fermi level is pined in InAs/GaAs due to the presence of interface states [24]; so, the variation of diffusion barrier due to the deposition of InGaAs can be expressed roughly as $Q=a/L_{w}^{2}+b$, where *L*_w_ is the QW width, *a* and *b* are the parameters independent on the QW width. The solid line shown in Figure 2b is the best fit results using this equation. The good fit reveals the changing of surface potential origin from the variation of energy levels. The deviation of fitting results for thickness <2 ML that is due to the fitting equation is obtained based on the assumption of infinite well potential [25,26]; so, it is not accurate if the thickness is very low. As to the reason why the diffusion barrier becomes stable for the layer thickness above 5 ML, it might be due to the electron concentration accumulated at the interface becomes saturated, which occurs when the deposited In(Ga)As layer is much thicker than the accumulation layer [24]. 

**Figure 3:**
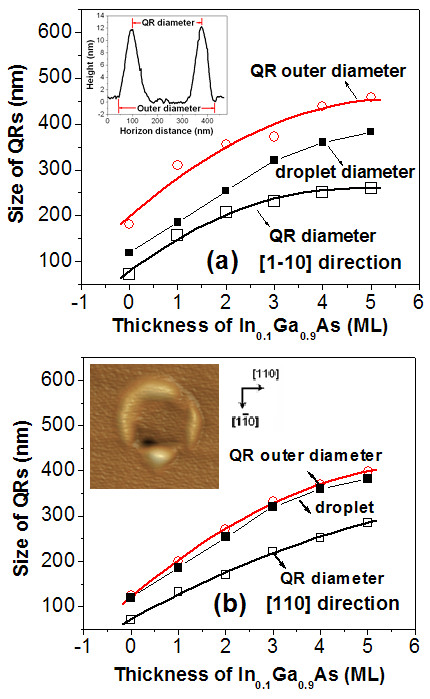
**Diameters of the QR.** QR's diameter (hollow sqares) and outer diameter (hollow circles) in (**a**) [1-10] and (**b**) [110] directions as functions of the thickness of In_0.1_ Ga_0.9_As layer.

**Figure 4:**
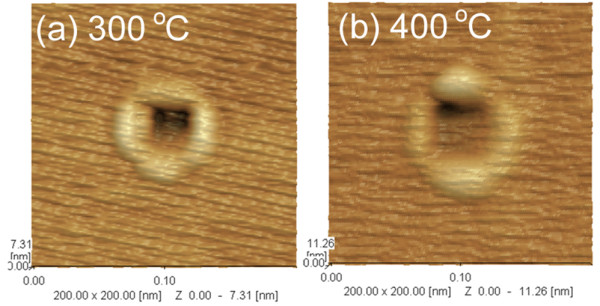
**AFM images of QRs.** QRs grown at (**a**) 300°C and (**b**) 400°C.

The variation in diffusion barriers will affect the size of droplets and, hence, the configuration of QRs. The measured diameters of QRs formed by annealing the gallium droplets in As4 ambient on different thickness InGaAs are plotted in Figure 3. Figure 3a,b shows the outer diameter (hollow circles) and diameter (hollow squares) of QRs in the [1-10] and [110] direction, respectively. The outer diameter and diameter are defined as the distance between the outermost and the highest position of ring wall (see the inset in the Figure 3a), respectively, and can be used to describe the diffusion distance of gallium atoms. The droplet diameter (solid squares) is presented as the reference line. The inset in Figure 3b shows the AFM image of single QR formed on the 5-ML In_0.1_ Ga_0.9_As. As can be seen, the QRs reveal the marked asymmetry in the [1-10] and [110] direction. The outer diameter in [1-10] direction is much larger than the droplet diameter; in contrast, the outer diameter in the [110] direction is just slightly larger than the droplet diameter, which demonstrates that the gallium diffusion in As4 ambient is dominated by the diffusion in [1-10] direction. All the diameters of QRs increase with the increase in the thickness of InGaAs and tend to be unchanged in the [1-10] direction for the InGaAs layer above 5 ML. Note that the QR shown in the inset of Figure 3b shows the significant asymmetry, which is mainly due to the asymmetric diffusion of Ga in the [110] and [1-10] directions in the arsenic ambient [18]. The further growth experiments reveal that this asymmetry can be improved by selecting the low growth temperature, which are shown in Figure 4. As can be seen, the asymmetry of QRs grown at 400°C with density of 10/μm^2^ is much more evident than those grown at 300°C , which also indicates that the growth temperature has a great influence on the symmetry of Ga diffusion in different crystal directions in arsenic ambient. 

**Figure 5:**
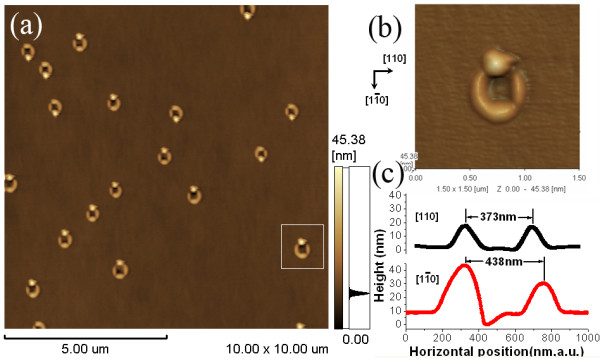
**AFM image of QR and its morphology.** (**a**) AFM image (10 × 10 μm^2^) of 15-ML QR grown on 14-ML In_0.1_ Ga_0.9_As layer. (**b**) the corresponding morphology of single QR, and (**c**) the cross-sectional profiles of QR in the [110] and [1-10] directions.

From the above discussion, it is found the QR size is mainly determined by the size of droplet, and the thick InGaAs layer means the large size QRs. So the gallium atoms equivalent to form 15-ML GaAs is deposited on 14-ML In_0.1_ Ga_0.9_As layer and annealed at 520°C in order to form the large size QRs. Figure 5a shows the AFM image (10 × 10 μm^2^) of the QRs. The morphology of a single QR shown in Figure 5b reveals that the QR consists of a nearly perfect ring wall and an island, which is just like the real jewelry ring. The scanned size shows that the diameter of QR in the [1-10] direction is about 438 nm, and the corresponding outer diameter is as wide as 736 nm. In the [110] direction, the QR's diameter is 373 nm and the outer diameter is 575 nm. Of course, the larger size QRs are also possible to achieve if the deposited amount of gallium, growth temperature, or the indium concentration in the InGaAs layer continues to be increased. 

## Conclusions

In summary, we had demonstrated the controlling of SA GaAs QRs' configuration by the QSE of InGaAs layer. By varying the thickness of InGaAs epitaxy layer underneath QRs, a drastic change of the shape, size, and density of the QRs was observed. It had been shown that the nucleus diffusion barrier can be decreased 14%, and the QR density can be lowered down to one QR per 6 μm^2^. Using this approach, the large size QR with the diameter of 438 nm and outer diameter of 736 nm had been achieved. We believed these results will contribute to the development of QR material and devices.

## Competing interests

The authors declare that they have no competing interests.

## Authors' contributions

TC performed the growth of QRs, did the measurements, and analyzed the data. YSF and WL coordinated the study. All authors drafted, read, and approved the manuscript.

## Authors' information

TC and WL are the professors of Changchun Institute of Optics, Fine Mechanics and Physics (CIOMP), Chinese Academy of Sciences (CAS), China. YSF is a professor in the School of Electrical and Electronic Engineering, Nanyang Technological University, Singapore.
